# Unusual Verrucous Skin Lesion in Diabetic Neuropathy Following Toe Amputation and Skin Grafting

**DOI:** 10.7759/cureus.69695

**Published:** 2024-09-19

**Authors:** Takahiro Kobayashi, Shin Iinuma, Akiko Watanabe, Akemi Ishida-Yamamoto

**Affiliations:** 1 Dermatology, Japanese Red Cross Kitami Hospital, Kitami, JPN; 2 Dermatology, Asahikawa Medical University, Asahikawa, JPN; 3 Plastic and Reconstructive Surgery, Japanese Red Cross Kitami Hospital, Kitami, JPN

**Keywords:** diabetes mellitus, diabetic foot, diabetic neuropathy, skin graft, toe amputation

## Abstract

Verrucous skin lesions in diabetic neuropathy (VSLDN) are rare and typically occur at weight-bearing sites due to chronic mechanical stress. This case report describes a 35-year-old man with type 2 diabetes mellitus who developed a verrucous lesion on the dorsum of his left foot following toe amputation and skin grafting. Histological analysis identified pseudoepitheliomatous hyperplasia with no evidence of malignancy or human papillomavirus infection. The lesion gradually resolved with the application of maxacalcitol ointment and the use of elastic stockings. This case highlights the importance of recognizing atypical verrucous lesions in patients with diabetes, particularly at non-weight-bearing sites after surgical interventions. Accurate differentiation between plantar warts and verrucous carcinomas is essential for appropriate management. Effective treatment and comprehensive patient education on foot care are crucial for improving outcomes and preventing further complications in patients with diabetes.

## Introduction

Diabetic foot lesions commonly arise due to peripheral neuropathy and peripheral arterial disease, conditions that are prevalent in patients with diabetes [1]. Sensory neuropathy results in the loss of protective sensation, motor neuropathy leads to foot deformities, and autonomic neuropathy causes skin dryness. These changes often contribute to the formation of calluses and ulcers, particularly at weight-bearing sites subjected to chronic mechanical stress.

Verrucous skin lesions in diabetic neuropathy (VSLDN) are characterized by hyperkeratotic, wart-like surfaces [2] and typically develop at weight-bearing locations such as the first metatarsal head, the plantar surface of the big toe, and the calcaneal region [3]. The case reported here is unusual, as it involves the development of a verrucous lesion at a non-weight-bearing site following toe amputation and skin grafting in a patient with diabetes.

## Case presentation

A 35-year-old man with a verrucous skin lesion on the dorsum of his left foot, which developed approximately two months after a split-thickness skin graft. Five months prior, his third toe had been amputated due to diabetic gangrene. The patient had a three-year history of poorly controlled type 2 diabetes mellitus, with complications including retinopathy and nephropathy. At the time of diagnosis, his hemoglobin A1c level was 14.3%. He was receiving insulin and metformin for diabetes management.

**Figure 1:**
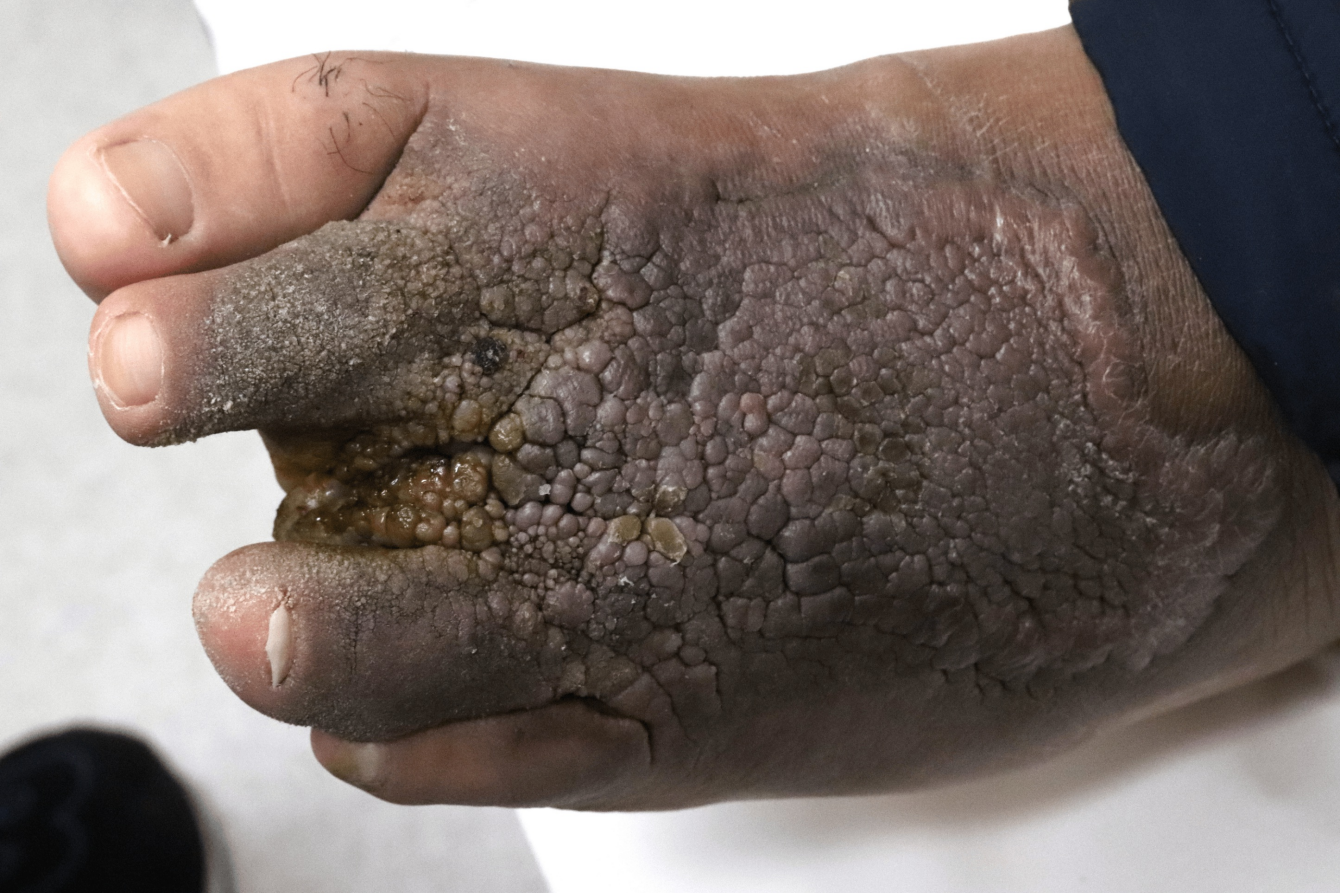
Clinical presentation Verrucous plaque with hyperpigmentation on the dorsum of the left foot, including the second and fourth toes.

**Figure 2:**
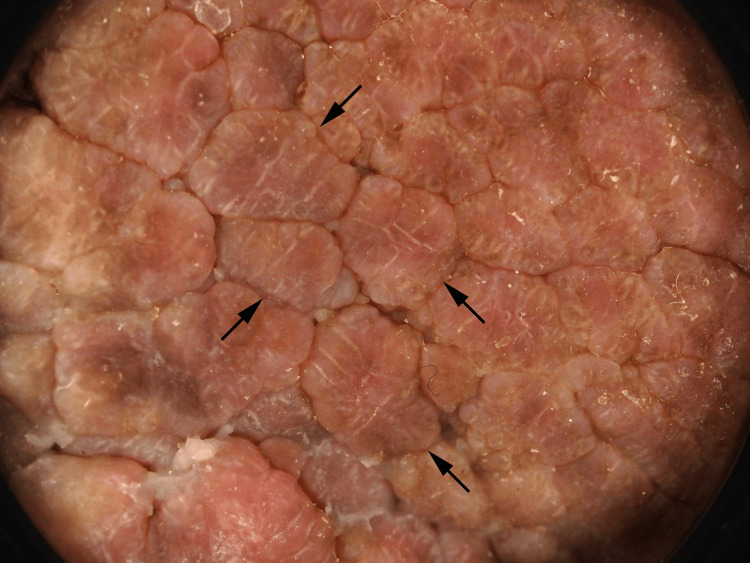
Dermoscopy of the lesion Cerebriform convolutions with densely packed exophytic papillary structures (arrows), without vessels or hemorrhages.

**Figure 3:**
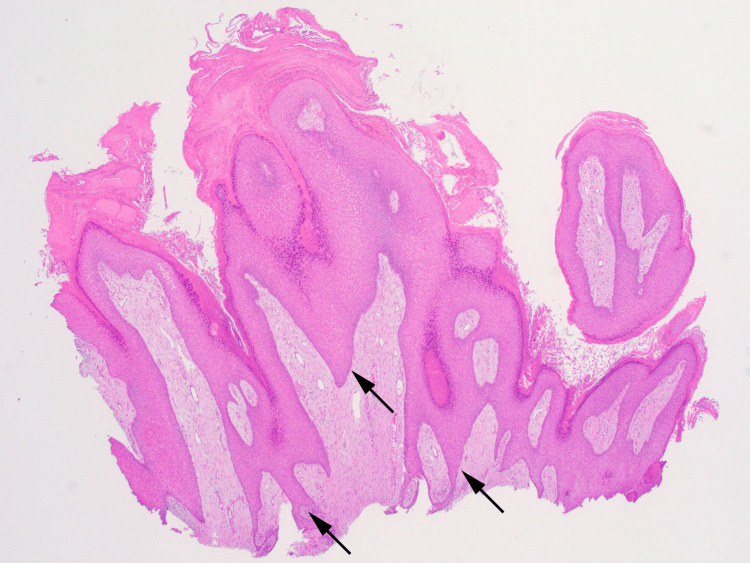
Histopathology of the lesion (low magnification) The lesion displays characteristic features of pseudoepitheliomatous hyperplasia, including epidermal hyperplasia, elongation of the rete ridges (arrows), and infiltration of inflammatory cells (hematoxylin-eosin staining; original magnification ×20).

**Figure 4:**
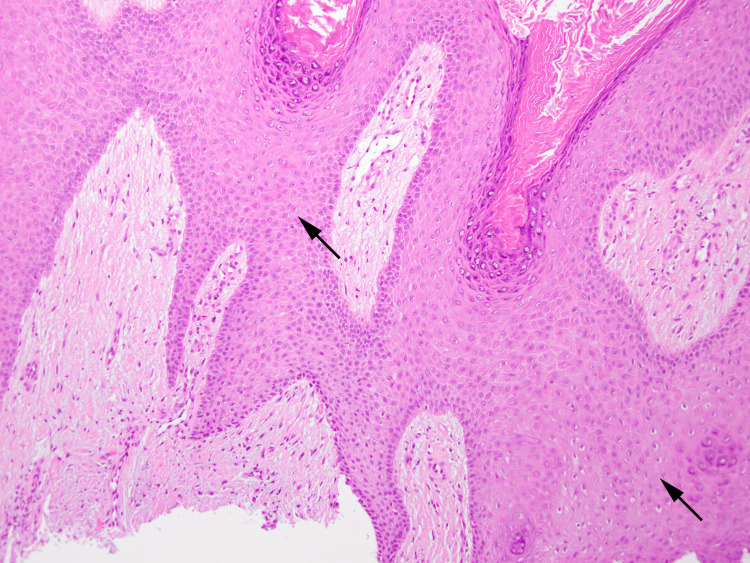
Histopathology of the lesion (high magnification) No findings suggestive of malignancy were observed (arrows), including nuclear atypia or individual cell keratinization (hematoxylin and eosin staining; original magnification ×100).

On physical examination, a verrucous plaque with hyperpigmentation was observed on the dorsum of his left foot, extending to the second and fourth toes (Figure 1). Additionally, calluses were noted on the plantar surface of the left foot. A neurological examination using a 5.07 monofilament confirmed sensory neuropathy in both feet. Dermoscopy revealed cerebriform convolutions with densely packed exophytic papillary structures, without evidence of vessels or hemorrhages (Figure 2). Histological examination showed epidermal hyperplasia, elongation of the rete ridges, and infiltration of inflammatory cells, consistent with pseudoepitheliomatous hyperplasia (Figure 3). No signs of malignancy, such as downward proliferation of the epidermis, nuclear atypia, or individual cell keratinization, were observed (Figure 4). Immunohistochemical analysis did not detect human papillomavirus antigen.

**Figure 5:**
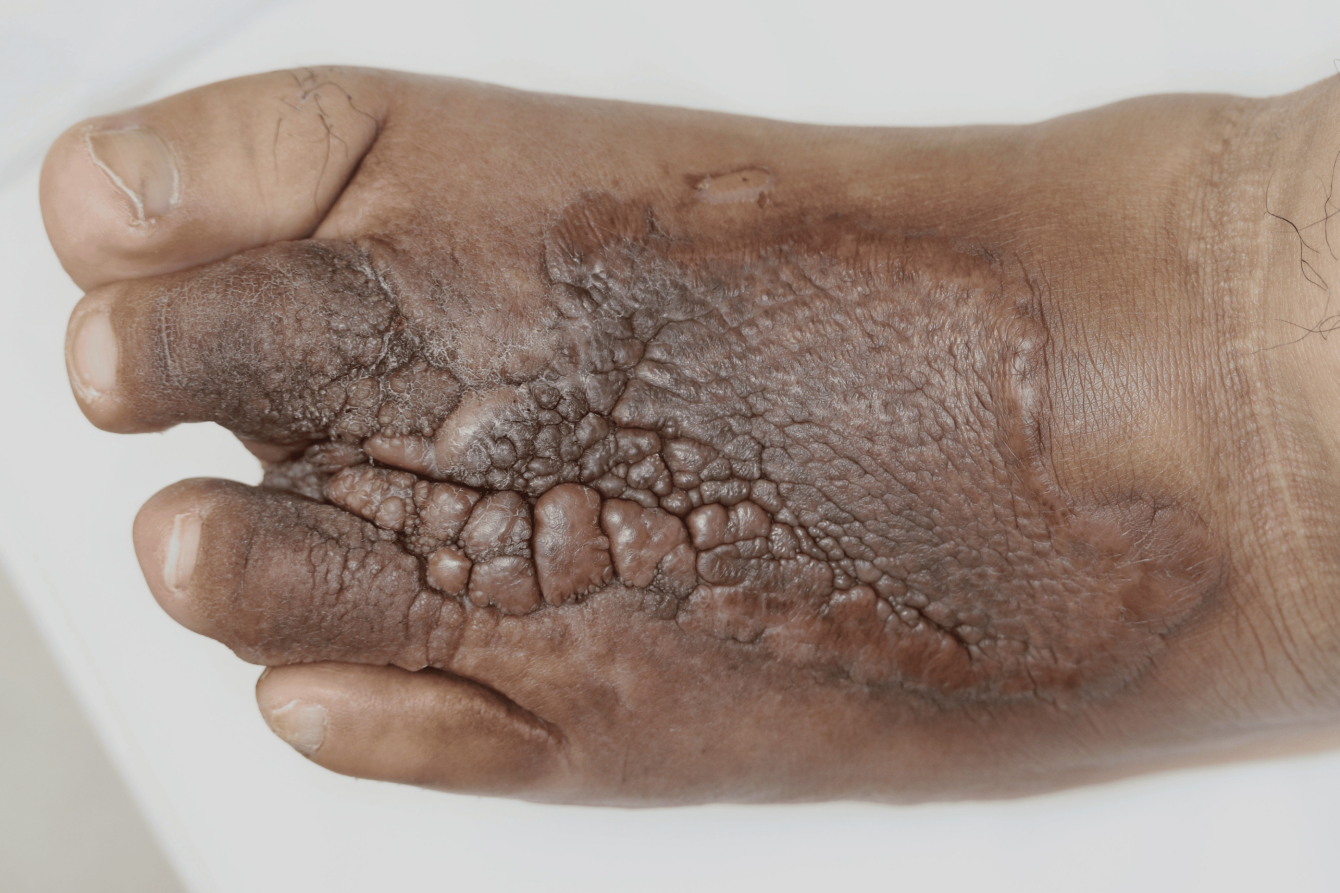
Clinical presentation post-treatment Two months after therapy initiation, a notable reduction in verrucous lesions was observed, although the granular appearance persisted.

Based on these clinical and histological findings, the lesion was diagnosed as a VSLDN. The patient was treated with maxacalcitol ointment and elastic stockings. After two months of treatment, the verrucous lesions reduced significantly, although their granular appearance persisted (Figure 5).

## Discussion

VSLDN is a rare complication that typically arises at weight-bearing sites such as the first metatarsal head, plantar surface of the big toe, and calcaneal region [4]. The exact pathogenesis of VSLDN remains unclear, but chronic mechanical stress, such as friction or pressure, is thought to play a significant role. Additionally, VSLDN has been reported in areas of scar tissue and skin grafts [5]. In this patient, the loss of the third toe due to diabetic gangrene likely caused chronic focal pressure on the dorsum of the left foot, contributing to the development of the lesion.

VSLDN can present in various forms: hard forms with verrucous hyperkeratotic lesions, soft forms with soft ciliary papillae, and combined forms that exhibit features of both [6]. In this case, the lesion exhibited atypical features, including dense papillomatous changes and less hyperkeratosis than previously reported. Despite the common occurrence of diabetic foot lesions, VSLDN is rarely reported and may be mistakenly diagnosed as a callus.

Histologically, VSLDN is classified as pseudoepitheliomatous hyperplasia, characterized by the proliferation of keratinocytes due to chronic inflammatory stimuli. Differentiating VSLDN from other conditions, such as plantar warts and verrucous carcinoma (VC), is crucial. Plantar warts and VSLDN, as both are predominantly located in areas of pressure and characterized by thick layers of keratotic material. In addition, both diseases were more prevalent in patients with diabetes mellitus [7]. The observation of multiple pinhead-sized bleedings suggested a diagnosis of a viral wart. Furthermore, dermoscopy revealed coiled vessels in cases of viral warts, which were not observed in the present case. Histopathological examinations, including immunohistochemistry for the human papillomavirus antigen, are also useful for differentiating viral warts from VSLDN.

VC is a rare low-grade variant of squamous cell carcinoma that presents as a slowly enlarging, warty tumor [8]. VC typically develops in areas of chronic irritation and inflammation and is most commonly observed in the feet. VSLDN and VC have clinically similar presentations, making it difficult to distinguish one from the other [9]. Histologically, VC shows a marked tendency to compress and displace deeper tissues through downward epithelial growth, although cytological atypia may not be conspicuous. In the present case, the verrucous skin lesions did not exhibit downward growth and displayed benign cytological characteristics.

Pseudoepitheliomatous hyperplasia is also observed in other conditions, such as lower limb lymphedema [10]. In this case, it was important to consider the diagnosis of elephantiasis nostras verrucosa, a rare complication of longstanding chronic lymphedema. Elephantiasis nostras verrucosa typically presents as diffuse, non-pitting edema and hyperkeratotic papulonodular with a verrucous appearance in the lower extremities. A previous report described the successful treatment of VSLDN associated with lymphedema using elastic stockings. Alleviation of VSLDN by elastic stockings indicates that the chronic inflammation caused by lymphedema may have been an exacerbating factor in the present case.

Treating VSLDN is challenging, as there is no established standard of care. Various treatment modalities have been reported, including repeated shaving of keratotic lesions, cryotherapy, topical treatments such as 5-fluorouracil, tretinoin, or vitamin D3 analogs such as maxacalcitol [11,12]. However, surgical intervention is generally not recommended. Preventative measures, including daily foot care and the selection of appropriate footwear, are crucial in managing VSLDN [13]. In this case, the patient responded well to maxacalcitol ointment, elastic stockings, and comprehensive foot care education, highlighting the importance of a multidisciplinary approach to managing this condition.

## Conclusions

This case report described a patient with diabetes who developed a verrucous skin lesion at an unusual non-weight-bearing site following toe amputation and skin grafting. VSLDN, while rare and typically found in weight-bearing areas, can also manifest in non-weight-bearing regions after surgical interventions. Recognizing and accurately differentiating VSLDN from conditions such as viral warts and VC is crucial for proper diagnosis and management. Early identification and appropriate treatment of VSLDN are essential for improving patient outcomes and preventing complications in individuals with diabetes.
